# Assessment of the RIFLE criteria for the diagnosis of Acute Kidney Injury; a retrospective study in South-Western Ghana

**DOI:** 10.1186/s12882-016-0318-3

**Published:** 2016-07-26

**Authors:** Richard K. D. Ephraim, Kwame O. Darkwah, Samuel A. Sakyi, Mabel Ephraim, Enoch O. Antoh, Prince Adoba

**Affiliations:** 1 Department of Medical Laboratory Technology, School of Allied Health Sciences, College of Health and Allied Sciences, University of Cape Coast, Cape Coast, Ghana; 2 Department of Molecular Medicine, School of Medical Sciences, College of Health Sciences, Kwame Nkrumah University of Science and Technology, Kumasi, Ghana; 3 Kumasi Nursing and Midwifery Training College, Kumasi, Ghana

**Keywords:** Acute kidney injury, RIFLE criteria, eGFR criteria, SCr/ePCr criteria

## Abstract

**Background:**

Acute kidney injury (AKI) affects 3–7 % of patients admitted to the hospital and approximately 25–30 % of patients in the intensive care unit. RIFLE, a newly developed international consensus classification for AKI, defines three grades of severity—class R (risk), I (injury) and F (failure). The aim of this study was to evaluate whether the RIFLE system of classification can detect the incidence of AKI using retrospective data of in-patients at the Effia-Nkwanta Regional Hospital.

**Methods:**

A total of 1070 in-patients’ records spanning a period of 6 months, from July 2014 to December 2014, was used. Demographic data and hospital admission serum creatinine of each participant were used for the calculation of estimated glomerular filtration rate (eGFR) using the 4-variable modification of diet in renal disease (MDRD) equation. Also, the baseline serum creatinine was estimated assuming a standard GFR of 75 ml/min/1.73 m^2^ using the simplified MDRD equation.

**Results:**

Males had higher serum creatinine, eGFR, and baseline serum creatinine than females (*P* < 0.0001). However, the level of increase in baseline serum creatinine was higher in females than males (*P* = 0.0212). The percentage ratios of the various classes from the SCr/ePCr (hospital admission serum creatinine/estimated plasma creatinine) criteria (R-1.45, I-1.53 and F-3.26) were higher than that of the eGFR criteria (R-0.34, I-0.11, F-0.12). The SCr/ePCr criteria gave more risk (89.7 %) than that of the eGFR criteria (23.1 %). The number of Injury and normal patients from the eGFR criteria was higher than the SCr/ePCr criteria.

**Conclusion:**

AKI was common in the ICU population with SCr/ePCr detecting more AKI than the eGFR criteria. Males had more injury and failure than females using the eGFR criteria whereas the SCr/ePCr gave females more risk and injury than males. A prospective cohort study must be employed in subsequent studies using the RIFLE criteria to assess the incidence of AKI in hospitalized patients with known diseases or medical conditions.

## Background

Acute kidney injury (AKI) is classically defined as an abrupt and sustained decrease in renal function [1–3]. However, there is neither agreement on the best method of assessing renal function nor consensus on the exact cutoffs for the diagnosis of AKI. This challenge not only leads to conflicting reports in literature but is also believed to be a major obstacle to research in this field [2, 4, 5].

There are no reliable estimates of AKI incidence across Africa [6], however, recent studies from Uganda report an AKI prevalence of 16.3 among sepsis patients [7]. But, findings from studies from other parts of the world indicate that AKI is common especially among hospitalized patients and affects some 3–7 % of patients in hospitals and 25–30 % of intensive care patients [8]. AKI ultimately results in morbidity, or will consequently demand the use of renal replacement therapy procedures such as dialysis or will result in mortality [9–11].

AKI in sub Saharan-African countries including Ghana is mostly attributed to infections like HIV/AIDS, malaria, diarrhoeal diseases; nephrotoxins and obstetric and surgical complications [12–14]. Also AKI in Ghana has been associated with the use poorly prepared herbal preparations [12]. Consequently, the International Society of Nephrology (ISN) introduced the ISN 0 by 25 to reduce the occurrence and impact of AKI [15].

A standard way of diagnosing AKI has been introduced with the acute kidney injury network (AKIN), RIFLE, and the kidney disease improving global outcomes (KDIGO) guidelines [16, 17] to ensure effective diagnosis and proper management [16]. The Acute Dialysis Quality Initiative (ADQI) formulated the Risk, Injury, Failure, Loss, and End-stage Kidney (RIFLE) classification for the diagnosis of acute kidney injury [18]. RIFLE, an international consensus classification for acute kidney injury, defines three grades of severity—risk (class R), injury (class I), failure (class F) and two outcome classes (loss and end-stage kidney disease).

The evaluation of renal function in people with suspected acute renal failure (ARF) or AKI is based solely on urinalysis, urea and creatinine estimation which cannot predict the severity of the renal impairment. However, studies in Europe and Australia have established the use of the RIFLE criteria in the diagnosis and subsequent management of people with AKI [19, 20]. Despite its increasing incidence, AKI in our setting [13] is still diagnosed by the estimation of serum creatinine. For this reason we chose to use the RIFLE criteria to access AKI among Ghanaian patients. This we believe will enhance monitoring of the progression of acute kidney Injury severity and possibly restoration of renal function in hospitalized patients.

Thus this study sought to use retrospective data of ICU patients in the Efia Nkwanta Regional Hospital to determine the incidence of AKI, and access whether it is feasible to use the RIFLE criteria in this context. This will be achieved by assessing AKI among in-patients under the estimated glomerular filtration rate (eGFR) and the increase in factor (SCr/ePCr) criteria and also comparing the distribution of AKI among gender from the eGFR and the SCr/ePCr criteria.

## Methods

### Study setting/study design

This was a retrospective single-centre study conducted from January 2015 to April 2015 among in-patients at the Intensive Care Unit (ICU) of the Effia-Nkwanta Regional Hospital (ENRH) in the Western Region of Ghana. The hospital offers both general and specialist care services in internal medicine, general surgery, paediatrics, obstetrics and gynaecology, dental and eye care and serves as the main referral facility for the western parts of the country. The hospital admits over 7500–10,000 patients annually. The ICU of the ENRH mostly admits patients with obstetric, medical (sepsis), surgical complications, victims with severe trauma from road traffic and other accidents and patients with snake bites.

### Participants/collection of data

Retrospective data of 1070 ICU patients (447 males and 623 females) were obtained manually from the laboratory database. Data of in patients admitted from July to December 2014 (due to completeness of data within period) were included whereas data of the following participants were excluded: patients less than 18 years and above 70 years, (since the MDRD in RIFLE criteria is valid for people within these age range), patients who are on dialysis and also kidney transplant patients.

Demographic and laboratory data of patients were retrieved from the laboratory database. Creatinine concentration on admission which was measured by the modified Jaffe’s techniques was used to calculate the glomerular filtration rate by using the Modification of Diet in Renal Disease (MDRD) equation [21].

$$ \mathrm{G}\mathrm{F}\mathrm{R} = 175 \times {\mathrm{SerumCr}}^{\hbox{-} 1.154} \ast {\mathrm{age}}^{\hbox{-} 0.203} \ast 1.212\ \left(\mathrm{if}\ \mathrm{patient}\ \mathrm{is}\ \mathrm{black}\right) \ast 0.742\ \left(\mathrm{if}\ \mathrm{female}\right). $$

According to the RIFLE criteria, the decrease in percentage of the calculated GFR must be >25, >50 or >75 % as compared to the standard (ADQI—Acute Dialysis Quality Initiative). The MDRD equation was used to calculate the Glomerular Filtration Rate (GFR).

ADQI and thus the RIFLE criteria recognized that in the acute situation, measured baseline creatinine is not always available for all patients [22–24]. Consequently, they recommended that an estimated baseline creatinine be calculated using the Modification of Diet in Renal Disease (MDRD) formula with an assumed GFR for all patients between 75 and 100 ml/min [25].

Estimated serum creatinine was calculatedwith the MDRD formula uses the following equation:

$$ epCr={\left(\frac{GFR}{Sex\times Race\times 186\times Ag{e}^{-0.203}}\right)}^{-\frac{1}{1.154}}\left(\mathrm{mg}/\mathrm{dl}\right) $$

Where *GFR* is the assumed GFR (75 ml/min/1.73 m2); *Sex* = 1 if male and 0.742 if female; *Race* = 1.21 if black, otherwise *Race* = 1; and *Age* is in years.

The ADQI recommendation is to assume that patients with a previously unknown renal function with no known kidney disease have a GFR of 75–100 ml/min/1.73 m^2^, and then to back-calculate the SCr value using the simplified MDRD equation. Hence, this study employed the simplified MDRD equation to back-calculate the baseline serum creatinine (SCr) assuming a glomerular filtration rate of 75 ml/min/1.73 m^2^ for the calculation of the baseline serum creatinine.

With a constant GFR and race, variable values for sex and age, the different baseline serum creatinine concentrations were calculated for each patient using the above equation and then compared with the hospital admission creatinine to determine the increase in fold of the baseline creatinine. Therefore, to estimate for the increase in fold of the baseline, the hospital admission creatinine concentration was divided by serum baseline concentration.

That is *A* = SCr/ePCr where A is the increase fold, SCr = hospital admission creatinine concentration and ePCr calculated serum creatinine concentration. After a careful estimation of the percentage decrease in the eGFR and the increase in the amount of fold of the calculated serum creatinine, the patients were then classified under the various classes in the RIFLE criteria.

### Statistical analysis

Data was entered into Microsoft Excel and statistical analysis was carried out using Statistical Package for Social Sciences (SPSS) for Microsoft Windows, 13^th^ students’ edition. Continuous variables like age were reported using mean and standard deviation. Bivariate analysis was reported using t-test and multivariate analysis done using ANOVA and the significance level set at 0.05.

## Results

A total of 1763 data of ICU patients were sampled within the period from July to December 2014. Of these, 483 were below the age of 18, whereas the bio data for another 210 patients was incomplete. Thus, overall data of 1070 participants was used. Serum creatinine had been estimated for all of these participants on admission to ICU or when it became apparent that they had AKI and thus were referred to the ICU. Therefore, we calculated the estimated serum baseline creatinine for the purposes of this study.

Table 1 shows the demographic characteristics of study participants. In overall, 1070 participants were studied of which 47.29 and 52.71 % were males and females respectively. The highest and lowest frequency were found among the age groups 20–39 years and <20 years respectively. Most of the participants (82.06 %) were in age range 31–59 years.

**Table 1:** Demographic characteristics of participants

Variables	Frequency	Percentage
Age group		
< 20 years	24	2.24 %
20–39 years	447	41.78 %
40–59 years	431	40.28 %
60–79 years	168	15.70 %
Gender		
Males	506	47.29 %
Female	564	52.71 %

Table 2 shows the distribution of hospital admission serum creatinine, estimated glomerular filtration rate, back serum creatinine and the increase in baseline serum creatinine among gender. Males had higher SCr (*P* = 0.0001), eGFR (*P* < 0.0001), ePCr (*P* = < 0.0001) than females. However, females also had higher SCr/ePCr (*P* = 0.0212).

**Table 2:** Distribution of serum creatinine, estimated glomerular filtration rate, back serum creatinine, and the ratio of serum creatinine to back serum creatinine

Parameters	Males	Females	*p*-value
SCr (μmol/l)	101.20 (81.48–132.00)	83.85 (72.00–102.10)	0.0001
eGFR (ml/mins/1.73 m^2^)	92.32 ± 54.50	81.51 ± 30.07	<0.0001
ePCr (μmol/l)	121.20 ± 44.54	92.93 ± 6.54	< 0.0001
SCr/ePCr	0.80 (0.70–1.10)	0.90 (0.80–1.10)	0.0212

*P < 0.05, SCr = Hospital Admission Serum Creatinine, eGFR = estimated Glomerular Filtration Rate, ePCr = Back Serum Creatinine, SCr/ePCr = Ratio of Serum Creatinine to Back Serum Creatinine, μmol/l = micromole per litre*

The Table 3 shows RIFLE classification from the GFR and SCr/ePCr criteria. The percentage ratios from the SCr/ePCr criteria were higher as compared to that of the eGFR criteria.

**Table 3:** RIFLE classification from estimated glomerular filtration rate and baseline serum creatinine criteria and their respective percentage ratio

	eGFR criteria	% ratio	SCr/epCr criteria	% ratio
Risk	247 (23.08 %)	0.34	960 (89.72 %)	1.43
Injury	79 (7.38 %)	0.12	29 (2.71 %)	1.53
Failure	73 (6.82 %)	0.11	62 (5.79 %)	3.26
No AKI	671 (62.71 %)	1.00	19 (1.78 %)	1.00

*% ratio* Percentage Ratio, *No AKI* No Acute Kidney Injury

The number of Risk patients from the SCr/ePCr criteria was more than that from the eGFR criteria. However, the number of Injury and normal patients from the eGFR criteria were all higher as than the SCr/ePCr criteria.

Table 4 outlines the proportions of RIFLE classes from the GFR Criteria. Males had more injury (*P* = 0.06715) and failure (*P* = 0.1196) than females. Females had more risk per the same criteria than males (*P* = 0.0107).

**Table 4:** RIFLE classification from eGFR criteria

eGFR criteria	Total	Male	Female	*p*-values
Risk	247 (23.08 %)	99 (40.08 %)	148 (59.92 %)	0.0107
Injury	79 (7.38 %)	41 (51.89 %)	38 (48.10 %)	0.6715
Failure	73 (6.82 %)	41 (56.16 %)	32 (43.84 %)	0.1196
No AKI	671 (62.71 %)	325 (48.44 %)	346 (51.56 %)	0.6715

Table 5 shows the distribution of RIFLE classes among gender from the SCr/ePCr criteria. Females had more risk (*P* = 0.3222) and injury (0.2030) than males while failure was significantly higher in males than females (*P* =0.0071).

**Table 5:** Distribution of RIFLE classes among patients from the SCr/ePCr criteria

SCr/ePCr criteria	Total	Male	Female	*p*-value
Risk (0.5–1.9)	960 (89.72 %)	441 (45.94 %)	519 (54.06 %)	0.3222
Injury (2.0–2.9)	29 (2.71 %)	13 (44.83 %)	16 (55.17 %)	0.2030
Failure (>2.9)	62 (5.79 %)	37 (59.68 %)	25 (40.32 %)	0.0071
No AKI (0.2–0.4)	19 (1.78 %)	15 (78.95 %)	4 (21.05 %)	<0.0001

**Fig 1:**
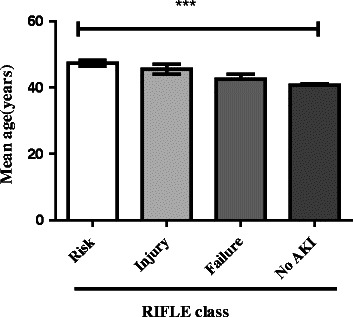
RIFLE classes against mean ages

Figure 1 summarizes the distribution of the mean ages in the RIFLE classes (AKI). Mean ages for males and females who had AKI were significantly higher than those who had no AKI (*P* = < 0.0001). The older people tended to get more Risk and younger people more Failure. There was an association between the mean ages and the type of RIFLE class (AKI) developed in the patient.

## Discussion

 This single-centre retrospective study used laboratory data to access the incidence of AKI among ICU patients in Ghana. We found that the RIFLE criteria detected AKI among ICU patients and SCr/ePCr (hospital admission serum creatinine/estimated plasma creatinine) gave females more risk and injury than males. In addition, mean age varied by RIFLE class with Risk tending to be associated with older mean age than Injury, Failure and no AKI respectively.

**Fig 2:**
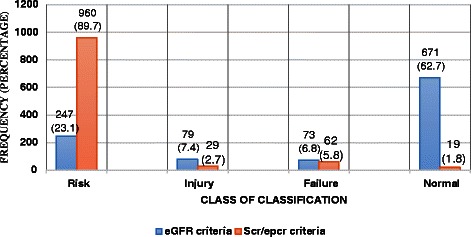
RIFLE classes from the eGFR and SCr/ePCr criteria

The occurrence of AKI in the patients from eGFR and the SCr/ePCr criteria were inconsistent with a prospective cohort study conducted by Macariello et al*.,* [26] in Brazil. This discrepancy could be due to the multi-center prospective cohort design employed, differences in variables selected (eg. old age and patients with three or more organ dysfunction) and the sample size (214) used in their study. From the RIFLE criteria when more than one sub-criteria is used on a single patient the patient is classified under the worst class [23] a finding confirmed by observations in this study (Fig. 2). This outcome ultimately helps in the early detection of patients at risk so that they can be effectively managed to prevent adverse outcomes.

However, the occurrence of AKI among patients from the SCr/ePCr criteria in this study was in line with that of Hoste et al*.,* [27] in a retrospective cohort study conducted at Pennsylvania on 5383 patients in seven intensive care units admitted during a 1-year period using the SCr/ePCr criteria. He and the colleagues found out that there was more AKI incidence than non-AKI among the studied population. The use of the SCr/ePCr criteria in this study gave a higher risk incidence which confirmed the Pennsylvania study [27].

The proportion of patients under the RIFLE classes from the SCr/ePCr criteria gave 89.7 % in R, 2.7 % in I, 5.8 % in F and 1.8 % in non-AKI contradicts the findings of an earlier study [28] in patients undergoing cardiac surgery, where there were 15.1, 9.1, 4.8, 71 % in the Risk, Injury, Failure and non-AKI classes respectively. The differences in the occurrence of AKI among the patients may be due to our inability to consider the disease condition(s) of the patients in this study, the kind of study design (prospective multicenter analysis) and the sample size used in earlier study.

Multi-centre and cohort studies [29, 30] in Canada also commented on the lack of linearity in using the eGFR criteria in the assessment of AKI in hospitalized patients. In this study, the use of the SCr/ePCr criteria gave more AKI in the patients (98.72 %) than the eGFR criteria (37.28 %) and confirms the findings of Ostermann et al*.,* [30].

The mean ages (years) of patients under the RIFLE classes were 47.4, 45.42 and 42.4 from class R to F respectively, 40.6 under the non-AKI category which showed that there was a significant correlation between the mean ages and AKI. The percentage ratio of RIFLE classes R to F from the eGFR criteria were 0.34, 0.12 and 0.11 respectively while that from the SCr/ePCr criteria were 1.43, 1.53 and 3.36 respectively. This depicts that the probability of an individual from the non-AKI group falling into any of the RIFLE classes from the SCr/ePCr criteria will be higher than that of the eGFR criteria. The use of this criterion will go a long way in providing solutions to the challenges associated with the diagnosis of AKI in the hospital setting. This will subsequently lead to the establishment of diagnosis so that the necessary interventions can be provided.

The strengths of our study is the fact that it is the first of its kind, to our knowledge, to assess AKI in sub Saharan Africa using RIFLE criteria or any of the guidelines (AKIN or KDIGO) to provide epidemiological data on AKI.

Our study however has a few limitations: first, the RIFLE criteria require urine sample measurement at specific time intervals to help in the classification of patients under the various strata of the criteria. The absence of urine in this study however will not be a problem since the other two estimated parameters (percentage decrease in eGFR and serum creatinine baseline increase) can equally help to evaluate AKI in hospitalized patients using the RIFLE criteria [31]. Second, even though the MDRD equation has not been validated in the Ghanaian population and used mostly in the assessment of CKD [32], the RIFLE criteria requires that this equation be used in the assessment of AKI and also as with many AKI studies, the absence of baseline serum creatinine means that estimation of these values was required to define AKI. Finally, the use of a single centre, a retrospective design, limited descriptive information of participants, potential sources of chance, bias and confounding in their findings will also limit the scope of our outcomes.

However, in a situation where there is no correlation between the eGFR and the measured urine in classifying AKI patients under the RIFLE criteria, preference is given to the eGFR since it best predicts the function of the kidneys [31].

## Conclusion

This study of use of RIFLE criteria to define AKI in hospital ICU population in Ghana using retrospective data analysis showed that AKI was common in the ICU population with SCr/ePCr detecting more AKI than the eGFR criteria. A prospective cohort study must be employed in subsequent studies when the RIFLE criteria is used to assess the incidence of AKI in hospitalized patients with known diseases or medical conditions. Further research should consider the determination of mortality rate in the various classes under the RIFLE criteria**.**

Research Implications of Findings: Our findings imply that the RIFLE criteria which aids in early detection and staging of AKI for better outcomes and management is useful in the Ghanaian population.
